# 1668. Antibiotic Usage Trends Following Implementation of Handshake Stewardship at a Tertiary Care Children's Hospital

**DOI:** 10.1093/ofid/ofad500.1501

**Published:** 2023-11-27

**Authors:** Lukasz Weiner, Sarah Auerbach, Patrick M Kinn

**Affiliations:** University of Iowa Hospitals & Clinics, iowa city, Iowa; University of Iowa Hospitals and Clinics, Iowa City, Iowa; University of Iowa Hospitals & Clinics, iowa city, Iowa

## Abstract

**Background:**

Antimicrobial stewardship was implemented at the University of Iowa Stead Family Children’s Hospital in November 2018 principally through handshake stewardship with pediatric intensive care and hospitalist teams. We sought to evaluate the impact of this intervention on the use of select antibiotics targeting *P. aeruginos*a and methicillin-resistant *Staphylococcus aureus* (MRSA).

**Methods:**

Use of select anti-pseudomonal (cefepime, piperacillin/tazobactam and meropenem) and anti-MRSA (vancomycin, linezolid and daptomycin) agents within our pediatric intensive care unit (PICU) and pediatric wards (L9 and L10) was retrospectively reviewed. Mean days of therapy (DOT) per 1000 patient days (PD) was aggregated and trended annually from January 2018 until December 2022. Simple linear regression analysis was conducted to detect significant changes in antimicrobial use over time.

**Results:**

Use of anti-pseudomonal and anti-MRSA agents in the PICU declined by 40% and 29%, an absolute reduction of 99 and 60 DOT/1000 PD, respectively (P-value: 0.0366 and 0.0210, respectively) (Figure 1). Use of anti-MRSA agents on L10 declined by 52% (absolute reduction of 53 DOT/1000 PD; P-value: 0.0263), but only declined by 6% on L9 (Figure 2, Figure 3). Use of anti-pseudomonal agents on L10 declined by 56% (absolute reduction of 44 DOT/1000 PD; P-value: 0.0522), but only declined by 10% on L9 (Figure 2, Figure 3).

**Figure 1:**
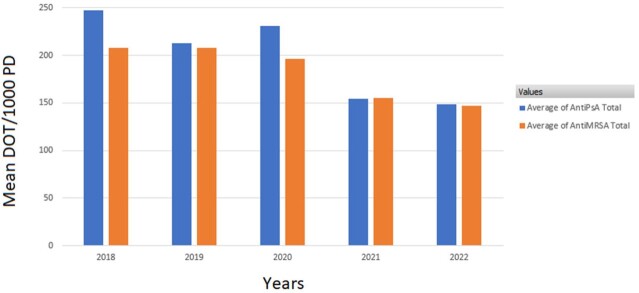
Mean Days of Therapy Per 1000 Patient Days of Anti-Pseudomonal and anti-MRSA Usage in the PICU

**Figure 2:**
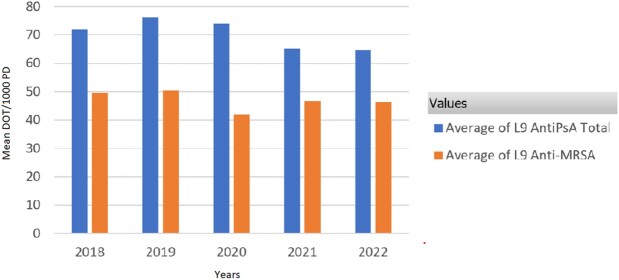
Mean Days of Therapy Per 1000 Patient Days of Anti-Pseudomonal and anti-MRSA Usage on L9

**Figure 2:**
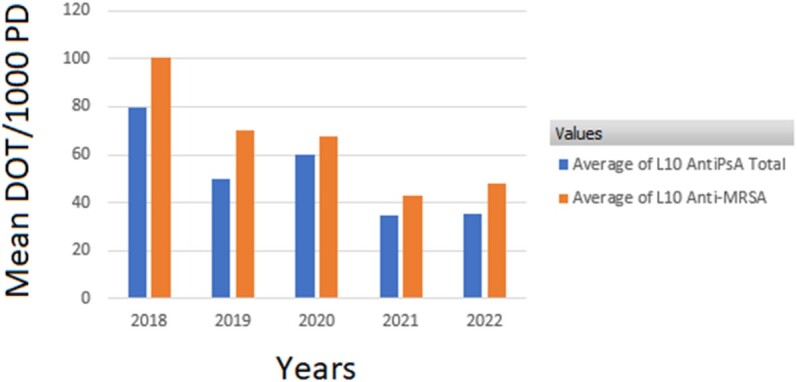
Mean Days of Therapy Per 1000 Patient Days of Anti-Pseudomonal and anti-MRSA Usage on L10

**Conclusion:**

Implementation of a handshake stewardship intervention strategy was associated with decreasing DOT of select antibacterial agents. The inability to significantly impact antimicrobial usage on L9 was potentially impacted by this being a mixed unit with subspecialty and surgical services. Expansion of handshake stewardship to subspecialty and surgical services may further reduce usage on mixed units.

**Disclosures:**

**All Authors**: No reported disclosures

